# Flagship Afterthoughts: Could the Human Brain Project (HBP) Have Done Better?

**DOI:** 10.1523/ENEURO.0428-23.2023

**Published:** 2023-11-14

**Authors:** Yves Frégnac

**Affiliations:** 1 UNIC-NeuroPSI, University Paris-Saclay, 91190 Gif-sur-Yvette, France; 2 Cognitive Sciences at Ecole Polytechnique, 91120 Palaiseau, France

**Keywords:** flagship, human brain, mesoscopic modelling, reverse engineering, simulation, virtual experimentation

## Introduction: Flagship Afterthoughts

Commenting about science has risks. Being critical sometimes raises strong opposing reactions. People work so hard and leaders do not like to see their strategies under fire. Critics do not usually provide easy solutions to the problems they raise, and the questions, even if they are right on target, remain largely unanswered. When the stakes are high and massive funds wait to be delivered, the train (or ship), once launched, ought not to derail (nor sink). It must go on, as planned, keeping the initial thrust alive.

In terms of management efficacy, a typical reason why the project’s leadership does not answer critiques is to “keep the monkey(s) on the critics’ shoulders” (Oncken and Wass, 1974; Cover, 1999). Being proactive may be a better way to get rid of the monkeys and open a constructive dialogue. The issue then becomes: what could have been done instead, for a better science? This is typically the question that I am asked at the end of my neuro-epistemological talks, or in comments received following prospective reviews on global neuroscience initiatives (Frégnac and Laurent, 2014; Frégnac, 2017, 2021).

My motivation to write about flagships and global neuroscience comes from my long involvement in interdisciplinary consortia, first as the biology coordinator for almost 15 years in successive European Future and Emerging Technology (FET) projects (Life-Like Perception, Bio-I3, Open-FET: Sensemaker, FACETS, Brain-I-Nets, BrainScaleS), then as an active participant in the ramp-up phase of the Human Brain Project (HBP). The present opinion paper is a commentary on the merits and limits of scientific strategies developed during the course of the HBP flagship. It should not be taken as an evaluation of the deliverables produced by eminent scientists and peers, with some of whom I have the pleasure and the honor to collaborate, or of the technological platforms created by the project, which I did not use. Accordingly, the focus here is not on individuals and their specific science, but on flagships, their metaphoric drive, their ups and downs in the making, and how they could serve the future of brain sciences.

## The Origin of the Flagship Concept

Let us rewind the film of the history of the Human Brain Project (HBP), back to 2011. More specifically, to the date of the contest between six flagship proposals organized by the FET initiative of the Information and Communication Technologies Directorate of the European Community (EC-ICT), before two projects, HBP, in the brain science field, and Graphene, in the 2D material physics domain, got lucky (Cramer and Hallonsten, 2020).

The initial lobbying of European flagships was motivated by several goals. The first was obvious, to benefit from the momentum of an unprecedented thrust in funding, fueled for 10 years (100 million euros per project per year). The second was to consolidate a research axis with a time span two or three times longer than classical funding schemes (10 years vs three to four years). The third was to develop “visionary, large-scale, science-driven research initiatives which tackle grand scientific and technological challenges across scientific disciplines” (Carrozza et al., 2017). Flagships were meant to be blue-sky projects, revolutionizing conceptual knowledge, addressing challenges thought to be at the limits of feasibility at the time, and generating disruptive technologies. In this context, the priority for HBP was to enforce a paradigm shift (Kuhn, 1962), that would revolutionize the way we look at the brain in terms of science and applications (Markram, 2006, 2012, 2013; Kandel et al., 2013). Note here that, in the context of the “blue sky” framing, delivering what had been promised was a different issue, and probably, in most minds, not mandatory. Indeed, in the case of HBP, most participants were convinced from the start of the project that the simulation of a fully digitized human brain was unlikely to be achieved in a 10-year span. The fourth goal was to foster interdisciplinarity in Europe and future emerging technologies. The new frontier targeted by HBP was at the interface between brain sciences and ICT fields, the hallmark of previous Bio-ICT and Neuro-IT proactive FET initiatives of the European Community (de Touzalin, 2013). The fifth was to increase the societal return of scientific research. For HBP, the obvious targets were the domains of public health (curing brain diseases), digital personalized medicine and brain-inspired ICT technologies.

The ranking among these motivations at the start of the project depended most likely on who you were, and what your primary domain of expertise was: a neuroscientist or a physicist in search of heavy funding to sustain risky research, a theoretician in search of the ultimate database to crunch, a computer scientist in need of exaflops, an IT entrepreneur in search of a new marketing bubble, an EC Eurocrat manager in charge of global policy design, a politician in search of a banner.

Other reasons played a role in the corridors of the European parliament, such as simplifying the scientific agenda of an entire field of innovative research, and pushing it to self-organize. In practice, projects in “big science” are veiled in a cloud of metaphors, raising the stakes of feasibility to the limit. “Building a digital brain” was the original motto of Henry Markram in 2011. “Virtual Brain” and “Digital Consciousness” have become the hottest HBP deliverables in 2023. The coming age of “Digital Medicine” is now the main target of Thomas Skordas, Deputy Director General of DG Connect, for the new European Horizon.

Central administrations and politicians indeed favor megaprojects that become the responsibility of a few charismatic scientific drivers. Their charge is not only to defend a common banner, but also to help organize the collective energies of an army of scientists to the service of technology-driven applications. For sure, the metaphoric semantic drive of the flagship has several virtues, serving: (1) to be readable and attractive to a large public, and strike the imagination by its boldness; (2) to bring industrial and private investors under a common flag; (3) to be vague and adaptive enough to support several interpretations (behind which administrators and Eurocrats can easily hide if the initial goals are not met…).

## Ten Years Later

So where do we stand now? Time has gone by since the initial promises, and the Human Brain Project (HBP) is wrapping up, after 10 years. Forget the buzz of the hot metaphors, which harm the public perception of the flagship. They created expectations that HBP scientists, despite their talents, could not reasonably meet. It is no surprise that some of us, including me, think that the final results are disappointing when considering the massive funding, and their innovative value appears somewhat downscaled relative to the initial claims.

Despite a dominant skepticism in the scientific community (Mullin, 2021; Sillig, 2023), some lukewarm (Deeg, 2023; Naddaf, 2023) and even laudatory (Hughes-Castleberry, 2023; Geiser, 2023) reports have accompanied the end of the European flagship initiative. One should acknowledge the scientific productivity of hundreds of talented scientists funded, at least partly, by HBP. In particular, impressive efforts have been made, in the last months of HBP, to document the legacy of HBP, both for the scientific community and the public. Beautifully presented syntheses and summaries of highlights give credit to 3000 publications and technical reports [website addresses and brochures are listed underneath.

- https://www.humanbrainproject.eu
- https://www.humanbrainproject.eu/en/science-development/scientific-achievements/highlights/
- https://www.humanbrainproject.eu/en/follow-hbp/news/2023/09/12/human-brain-project-celebrates-successful-conclusion/
- https://sos-ch-dk-2.exo.io/public-website-production-2022/filer_public/74/94/74948627-6a92-4bed-91e0-3fab46df511d/hbp_spotlights_achievements_2023.pdf
- https://sos-ch-dk-2.exo.io/public-website-production-2022/filer_public/6f/70/6f706305-a2e3-45b8-a42b-dfb476222a6a/230413_hpb22_digital.pdf
- https://sos-ch-dk-2.exo.io/public-website-production/filer_public/63/0d/630d6561-e26a-4e74-84bf-b5cbc63deab4/hbp_brochure_spotlights_digital_lowres.pdf

State-of-the-art atlases and impressive simulation showcases have been delivered, but the announced paradigmatic “rupture” remains to be demonstrated. Currently, at the end of HBP, scientists do think of the brain in “bigger” terms, the technologies have progressed remarkably, but the driving concepts in the fields of brain sciences and neuromorphic computing remain largely unchanged.

The assumed bias of this opinion paper is to focus on bottlenecks and strategical disappointments, following the rationale that one tends to learn more from failures than successes. Success means the strategy was right and predictable, and nothing needs to be changed at this point. Failure, in contrast, tells us that something went wrong on the way or that the driving ideas needed at least to be adjusted, if not contested. Flagship afterthoughts, thoughts after the flagship, open a re-evaluation phase. This phase should not divide scientists into proselytist “Pros” advocates and fierce “Against” critics. The flagship concept (and its implementation) merits a more balanced and scientific evaluation. So, let us look more in depth at what we did learn from HBP, in particular, the hurdles met along the path and the bottlenecks that could not be breached.

Four global observations come to my mind: (1) the difficulty of running an interdisciplinary project of unprecedented scale in brain sciences; (2) the difficulty of building a new community around a common agenda; (3) the fact that simulating the whole brain is a more complex task than expected, not only because of technological limitations, but also because of the lack of new concepts; (4) in fine, the difficulty (or impossibility) of building a comprehensive multiscale theory of the brain. Yes, the stakes were high, and it was probably worth a try, but more thoughts and attention should be given to understanding the reasons why, in the end, we are left with this awkward feeling of incompleteness.

## Building a Flagship *De Novo*

From a historical perspective, a difficulty for a European flagship specific to the interface field between brain sciences and ICT, was to define the starting point. With the exception of the European branch of the International Neuroinformatics Coordinating Facility (INCF), this was almost zero in terms of global shared infrastructure. Almost zero, in terms of global science management. Uncoordinated, in terms of completed databases. Fragmented, in terms of database ontologies. Elusively weak, in terms of integration across scales (from micro to macro). Lacking a coherent unifying theory. Weak and undecided, in terms of industrial investment intent.

The initial blue-sky feature of the HBP agenda, the one which won the flagship contest, was in sharp contrast with the translationally-driven orientation of the two other successful flagships, “Graphene” and, more recently, “Quantum,” for which industrial applications naturally prevailed. It is important to note that the content of the final document contracted by Henry Markram and EPFL with the EC in 2012 ended up being quite different from the one submitted for the flagship contest. The amended version strengthened the focus on databases and high-performance computing (HPC) infrastructures, and re-established the preeminence of IT over fundamental brain sciences. The restrictions on non-human primate (NHP) experimentation, which quickly followed, were decided without the unanimous consent of the board of scientific directors. These moves were part of the negotiation with the EC (from which most of the funding originated). They were most likely made to accommodate the IT-division directorate and reduce pressure from the European anti-vivisectionist lobby. In contrast, competing international mega-science projects in the field of brain sciences were, from the start, more technically targeted (Jorgenson et al., 2015; Huang and Luo, 2015; Grillner et al., 2016; Fairhall, 2021) and more experimentally focused [“record every spike in the brain” in Alivisatos et al. (2012); see the recent advances in the field in Kleinfeld et al., 2019; Demas et al., 2021].

The original task for HBP was thus quite unique and daunting. From the start, many neuroscientists and physicists thought that the challenge of targeting the full digitization of the Human Brain, presented as the ultimate “race-to-the-moon,” was not helping the field. Retrospectively, there also remains the doubt whether the scientific community in Europe was mentally prepared and mature enough, to engage in this type of unified enterprise, at such a scale.

### Building a new scientific community

A crucial hurdle, from the start, was that there was no solid census of scientists who would not only be interested enough, but who would also give their full adherence to a collective research roadmap. Science is often done by bright minds obsessed with private/individual narrowly-focused agendas. Apart from some notable mid-size FP7 and FET-Open initiatives (Daisy, BrainScales, Robocom), which gave rise to distinct and somewhat opposing lobbying (Dario et al., 2011; Markram et al., 2011; see also Martin, 2022), very few structuration efforts existed in Europe at a macroscale level before HBP. An attempt to consolidate scientific networks was made as early as 2004 by the National Bernstein Computational Neuroscience initiative. Its aim was to establish central nodes of excellence to strengthen German regional capacities in the field of computational neuroscience, interconnect them and develop applications in the neuro-IT and brain-machine interface domains. This structuring move was the result of a unique, large-scale funding initiative (in sum over 200 million euros) of the German Federal Ministry of Education and Research (BMBF), led with persistence by a remarkable officer, Christiane Buchholz (Schwarzwaelder and Cardoso de Oliveira, 2010; Biopro, 2011), and driven by ardent and dedicated scientists (Andreas Herz and Ad Aertsen, among others). This impressive success story, unique in the European scientific landscape in life sciences, should have inspired HBP. However, unfortunately for HBP, the internal conflict between theoreticians and Henry Markram, during the writing of the roadmap in 2011, led to the premature disengagement of the Bernstein centers and the United Kingdom Gatsby center(s) just before the start of the grant (Shtull-Trauring, 2012). An additional destructuring event occurred three years later, when the cognitive neuroscience pillar, directed by Stanislas Dehaene, was expelled from the main research axis by the triumvirate executive board coordinated by Henry Markram (an event recounted in Destexhe, 2021). This last move crystallized a deeper crisis between HBP and the neuroscience community (see the neurofuture letter coordinated by Mainen and Pouget, 2014; for review see Frégnac and Laurent, 2014; Mahfoud, 2021).

Did the flagship change the collective way we do brain sciences? Attracting and sustaining collaborations has been at the heart of the open calls of HBP (Lorents et al., 2023). Despite these efforts, monitored by ethnographic studies in social sciences (Mahfoud, 2018, 2021; Aicardi and Mahfoud, 2022; Rüland, 2023), it remains unclear at the end of the project what type of community has emerged or been consolidated. To my knowledge, no quantitative independent social network study has been yet made, looking at the full longitudinal history of the HBP flagship, to analyze (1) the evolution of collaborative networks (to illustrate for instance a possible revitalization by open calls) and (2) the interpenetration of the different scientific fields. Some of these networks, formed before HBP, had already been driving blue-sky projects (FACETS and BrainScaleS). These medium-sized interdisciplinary consortia became diluted in their merging with the partners of the Blue Brain Project (BBP; for review, see Frégnac and Laurent, 2014). The identification of fixed points (happy contributors constantly funded throughout the time course of HBP) is also missing. Did the funding mainly benefit preexisting scientific clusters, or sustain, from start to finish, a rich club of individual scientists? Did a new community of users of HBP infrastructures emerge in fine? My own impression is that the fixed points were tightly linked with the governance (EPFL, then Jülich) and that, around these, scientific subnetworks waxed and waned in succession. The consortium dynamics do not yet seem to have reached an equilibrium point where a stable envelope of labs could be constituted by regular users of HBP databases and digital infrastructures. This remains an open and fascinating issue for further social studies.

### Building consortium coordination

A more down-to-earth related issue is to assess how the coordination of the scientific communities targeted by flagships operates, and what mechanisms can be used to measure its effectiveness. In the case of Graphene, the coordination was led by the Department of Physics at the Chalmers University in Sweden. A decade later, it is clear that this large-scale initiative has globally succeeded in its endeavors. According to the final report, scientific and technological promises have been fulfilled. Sixty to seventy per cent of what was promised in terms of specific applications has been delivered. A quantified study led by WifOR (commissioned by the flagship) makes the projection that *Graphene* will have created a total contribution to GDP of €3800 million and 38,400 new jobs in the 27 EU countries between 2014 and 2030. Per euro invested and compared with other EU projects, this flagship has performed 13 times better than expected in terms of patent applications, and seven times better for scientific publications. Seventeen spin-off companies have received over €130 million in private funding (Albu and Juneja, 2023; see also “Flagship EC reports” given underneath.).

- The European Commission (DG Connect) and the GRAPHENE and the HUMAN BRAIN PROJECT FET Flagships (2016). FET Flagships. Lessons learned from the first 30 months of their operation.https://www.h2020.md/en/fet-flagships-lessons-learntfet_flagships-lessons_learnedfromramp-upphase-2016oct 25final_18843.pdf
- See also Public presentation by Wolfgang Boch, FET-Flagship information day, Bratislava, SK, 23 May 2013: “FET Flagship Initiatives: Concept, Call and Evaluation results”
- The European Commission (DG Connect) and the GRAPHENE and the HUMAN BRAIN PROJECT FET Flagships (2023). Ten years of the European Union’s roll of the dice: €1B or 1billion euros each for the Human Brain Project (HBP) and the Graphene Flagship. 09 September 2023.https://www.frogheart.ca/?tag=graphene-week-september-5-9-2022-is-a-celebration-of-10-years-of-thegraphene-flagshiphttps://graphene-flagship.eu/materials/news/chalmers-steers-europe-s-major-graphene-venture-to-success/

Related statistics and economic studies are still missing concerning HBP, and it is likely that the Brain flagship did not provide a similar return of investment, compared with its Carbon flagship companion.

Indeed, the Graphene project met all the expected evaluation criteria, mostly because it was purely technology-driven and primarily engineered and organized as an industrial project. Things operated differently in the HBP, because of its initial blue-sky ambition (see above), and also in its coordination. The EC chose to run the flagship through the prestigious Ecole Polytechnnique Fédérale de Lausanne (EPFL), led by the ambitious entrepreneur Patrick Aebischer. To put it frankly, at the time no other institution of similar experience volunteered in Europe to run the HBP flagship. The choice of EPFL was justified by the fact that two of the members of the scientific triumvirate leading HBP, the charismatic Henry Markram and Richard Frackowiak, were professors in this respected institution. However, in addition to the fact that EPFL belonged to a non-EC country, the risk existed of a possible conflict of interest, since the EPFL was already engaged in the management of the Blue Brain Project (BBP) with a strong United States industrial partner, IBM. The initial choice of a scientific writer as a general communication manager could also indicate that more attention was given in the ramp-up phase to the control of the wording of what could be done, rather than to the consolidation of the scientific network while keeping the objectives within reasonable reach. Multiscale, multiomic digital reconstruction of the brain became the flagged mottos. Hype was there, from the start.

In terms of coordinating administration and the validated assessment of achievements, HBP created an administrative stranglehold. Rather than capitalizing on the local administrative resources already present in the participating European institutions, an oversized centralizing administration effort was constructed ahead of the consolidation of any scientific project. Fifty-two high-salary administrative positions were filled at the start of HBP. Lessons still remain to be learnt. The same issue is still present today into the management of EBRAINS, in part with the same players. An overpowering administrative approach was repeated again at the start of EBRAINS, when several dozens of such positions were requested (unsuccessfully) in the framework of the Horizon-Infra-2022-SERV call. Despite 10 years of experience, a good managerial model is yet to be found.

An objective evaluation of the intrinsic weight of the management budget, together with indirect coordinating activities (relayed by the partnering institutions), is difficult to reconstitute. In HBP the official administrative share of the total funding allowance was initially estimated to be 7–8%, i.e., 70–80 million euros (Markram, 2012). This projected administrative share was equal to the global budget of the Theoretical Neuroscience pillar! On top of that, indirect costs and overheads financed by HBP meant that administrative funding operated as a multilayer skimming of the initial endowment, dependent on the national institutions: each participating institution’s administration engulfed a sizeable share of what was supposed to be scientific funding. The real numbers do not seem to be retrievable in a transparent way, and, strangely, Chat-GPT breaks down if you ask the question to Alice or its deep learning Ersatz!!

A third issue has been how to monitor the effectiveness of coordination. Taken globally, the HBP flagship productivity remains difficult to evaluate: many objectives were renormalized during the course of the project, and these were reviewed for the EC by a multiplicity of experts, who, themselves, were often renewed at each phase of the contract. These different factors contributed to the absence of a common evaluation grid, which would have allowed a coherent tracking overview, from start to finish. This lack of longitudinal continuity finds an echo in the fact that many of the scientists who participated in the early phase of the Human Brain Project, including Henry Markram, the founder of HBP, were not present at its closing ceremonies (the Summit meeting in Marseille in March 2023, open to the public, and the more introvert closing event, held in Jülich in September 2023).

Concerning the scientific job market in HBP, unexpected trends were observed. Because of the relative lack of trust which appeared after the mediation between the EC and the various individual state administrations, the deliverable reporting and the level of surveillance of the productivity of each postdoctoral researcher, engineer, and even animal care technicians, were increased. The famous Key Performance Indicator (KPI) metrics, which fit with the managerial monitoring of industrialized and financial projects (Twin, 2023), were probably not best adapted to track progress in the more fundamental aspects of HBP. This holds in particular for highly diverse brain research studies, where the validation process must take into account exploratory experimentation and where publication of results usually takes several years. In parallel, tracing follow-through continuity was aggravated by employment rules at certain national administrations, for example the universities and the Centre National de la Recherche Scientifique (CNRS) in France: to avoid risking the obligation to transform project-specific, short-term recruitment into permanent tenured positions, individual postdoctoral researcher employment was limited to a two-year span (in fear of the application of the French Sauvadet Law). A possible solution might have been found by allowing rotations of the hiring institutions, so that the national constraints, linked with a fixed employer, could be overcome. Opening the possibility that a researcher or technician working at a given site of the European consortium could be financed in turn by different administrations would have fitted well with the European dimension of the project. Despite the crucial need to secure funding of key staff members for up to 10 years, the surprising overall reality of the HBP job market was that employment contracts were on average shorter than in regular research grants, or even FET grants. The unproductive consequence was a loss of continuity in the work dependent on successive postdocs and high-level engineers. A reduction in scientific efficacy toward reaching the “hard science” objectives and a down-calibration in the ambitions of the deliverables resulted from this fragmentation in hiring opportunities.

### Building novel datasets

In terms of databases, considerable efforts in defining ontologies, including exhaustive analysis of metadata, are necessary ahead of any sharing of experimental work. It is only after agreeing on common classification labels that labs can interchange and convert their data around a common referential (Petilla Interneuron Nomenclature Group, 2008). Impressive attempts have been made beyond HBP, in particular by the Allen Institute (Koch and Reid, 2012), but large-scale initiatives have often kept their agenda and classification criteria separate, making a direct comparison difficult (Frégnac, 2017; Fairhall, 2021). In the case of HBP, no significant funding was allocated for the full implementation of interoperability of preexisting databases, including revisiting experimental lab notebooks according to accepted standards. This may have a cost in the long run: important structural and *in vivo* functional data, already acquired in species which have become out of fashion (cat, electric fish, giraffe.), might remain noncurated on some lab shelves. This difficult issue (no one wants to go back to old data formats) was already discussed between *in vivo* and *in vitro* neuroscientists at the time of exploratory projects of the FET initiative (Daisy, FACETS, BrainScaleS) in collaboration with the experts from the Blue Brain Project (BBP) at EPFL. Some aspects were partially explored by the INCF community (mostly neuroimaging and calcium imaging), leading to the widespread adoption of the Neurodata Without Borders format for new data acquisition and a solid semantic metadata framework (developed in the second half of HBP). It is however apparent at the beginning of EBRAINS (i.e., some 10 years later) that an unsatisfactory state remains, concerning mostly the functional levels of investigation where contextual metadata are impossible to reconstitute *post hoc*. For instance, still in 2023, the comparisons between functional *in vivo* and *in vitro* multiscale data remain inconclusive or superficial. It is fair to recognize that Henry Markram thought of this, and tried to annex INCF at the start of HBP, without much success at that time.

In terms of animal experimentation, HBP achievements have been limited by the contradiction between defining and implementing unified experimental paradigms, on the one hand, and using old data recorded in vastly different contexts, on the other hand. In principle, paradigmatic unification requires new experimentation sets. In reality, HBP limited the amount of animal experimentation in higher mammals (the species the closest to humans), focusing instead on rodents and tried to remove nonhuman primate (NHP) experimentation from its objectives, mostly for political correctness. Although this exclusion rule was relaxed in the second part of the grant, it greatly weakened interspecies comparisons and led to overreaching transfer of coding concepts, developed in rodents, to the human brain (Hodge, 2019; Lowe, 2019; Loomba et al., 2022).

By developing centralized database infrastructures, one benefit, foreseen by Henry Markram (Markram, 2012), was that the aggregation and mining of diverse long-tail data, as well as conversion of numerous small data sources into big data, would improve knowledge about neuroscience-related disorders (Markram, 2012; Ferguson et al., 2014). However, the shift in emphasis, during HBP, from efforts in data acquisition (mostly fine-scale multispecies physiology) and ontologies to the development of centralized database infrastructure (involving mostly exclusively neuroinformatics and HPC) and human neuroimaging had, in my view, an additional side-effect. Some governmental institutions in Europe (including France!) suggested that enough data may already be available on the laboratory shelves, constituting a pile of “siloed” dormant sources that just need to be curated (for review, see Choudhury et al., 2014; Frégnac, 2017). It seemed indeed easier in terms of budget control by institutions to turn scientists into high-tech engineers seeking patterns in existing data rather than to continue to fund basic research which requires new experimentation and costly animal care facilities. At the end of the road, the infrastructures for exploiting the datasets may be operational (Amunts et al., 2016), but what fraction of Europe’s neuroscience data has been curated to populate the promised HBP databases?

### Building a coherent scientific strategy

The major novelty introduced by HBP has been to promote the concept of “virtual experimentation” (Markram et al., 2011; Markram, 2012), already applied in medical studies of human organs and biomedicine [see VHP, the virtual human physiology project in FP7 (Hunter et al., 2013); for review, see Mulugeta et al., 2018]. The concept was generalized in HBP to “*in silico*” hardware platforms, where detailed neuromorphic simulations of biological experiments were interpreted by physicists as some kind of experimentation (see theoretical discussion in Hadky, 2019). Note that the motivation here was not completely scientifically driven, but provided an easy answer to societal doubts and legitimate concerns about animal vivisection. Indeed, switching research experimentation from *in situ* to virtual *in silico* experimentation certainly conforms to the three Rs of ethics guidelines favored by the EC (replacement, reduction, refinement; see Russell and Burch, 1959). However, in my view, giving scientific legitimacy to such a trend is here more hazardous than it seems, and its generalized practice could be misleading. Computer simulations and virtual experimentation are presented to the public as if they could replace animal experimentation. They do not.

HBP relates the concept of virtual experimentation to that of “predictive neuroinformatics,” i.e., the idea to predict new findings from previous ones without ever carrying out the experiments to support (or reject) these hypotheses. In essence, this leads to a body of data, some of which are real, others are predicted, and yet others that are predicted from predicted data etc. This strategy in HBP was meant to overcome a key issue in which, because of technical limitations, much needed experiments could not be conducted on all spatial/temporal scales. In the end, this strategy generated a conglomerate of fictive data, which may be related to the models from which they originate, but cannot be attributed to the biological system under study. The situation is different in physics, where theoretical models are strong enough to correctly predict experimental facts. This challenge may be one bridge too far in brain sciences.

Strictly speaking, “virtual” experiments are experiments that test new hypotheses on real biological data that were collected to test other hypotheses. Classically, the qualifier “virtual” is used to refer to data collected from existing datasets (for instance, from different labs) and collated into a unique data set rather than taken from a new set of animals (Peterson, 1995). This concept in HBP, and in deep learning models, has been extended to the read-out of sets of local internal “hidden” variables, which are the exclusive results of simulations for different contexts, without being systematically validated by the experimenter. In most cases, the global parameters of the model were initially defined by the specific context of the biological experiment. They were trained to fit the correlation matrix between recorded real data, i.e., external input, biological data recorded in sparse specific brain sites and the global output performance. The same model can thus be replayed for other classes of simulated inputs, and virtual responses can be predicted under various types of perturbations produced by modulatory-like or electrical-like artificial manipulations, or even following *in silico* lesions (Markram, 2014). This data-enriching approach has generated a lot of interest in the modeling field. It has also been applied with some success to psychology and neurolinguistics (Jain et al., 2023). In physics of materials, deep learning algorithms trained by extensive structure-property datasets have recently been optimized to predict the capability of generating new materials with targeted properties (Honda et al., 2021). This example of experiment-free strategy certainly represents important advances that will influence future research.

Complementary virtual experimentation strategies have been used in the second half of HBP course, adding an elaborate twist of sophistication. The initial concept of “The Virtual Brain” (TVB) dates from 2008 (Ritter et al., 2013; Sanz, 2013), well before HBP. By merging individual anatomy from brain imaging data with state-of-the-art mathematical modeling, its aim was to reduce complexity on the microscopic level aiming to reveal the macroscopic organization. The driving hypothesis is that a TVB model of a human patient’s brain activity generates sufficiently accurate EEG, MEG, BOLD, and SEEG signals to reduce the dynamic complexity by a million-fold through methods from statistical physics (Ghosh et al., 2008). This hybrid approach, already applied in human medicine, has been merged during HBP with the concept of “digital twin,” which originates from the engineering industry, especially aeronautics (Grieves, 2019). A “digital twin” is a type of personalized computational brain model, based on measured real-world data obtained from its real-life counterpart, i.e., the patient. This concept has been applied, with impressive success, by Viktor Jirsa and colleagues in predictive modeling of focal epilepsy (Jirsa et al., 2023). In this case, sensor data are recorded in the real world and then used to build a virtual model of the individual brain. However, by feeding the databases with virtual data simulated in a metaverse world, the scientific project tends to suffer from a kind of poor man’s logic (I cannot record for real, so I simulate). We are no longer studying the brain but the properties of its digital Ersatz, as if these were precisely identical at every level of description and function. Identical “twins”: can still one pretend?

In biological, neurologic, or psychological terms, we are very far from understanding the complexity of brain function: to what extent is the construction of a digital facsimile actually advancing knowledge of the human mind? Indeed, is there any reason to expect that digital models would, could or should achieve capabilities of function and adaptation (in the case of lesion/inactivation paradigms) identical to those of the real brain. What are the critical parameters in the virtual brain model that are necessary to substantiate the biological relevance of the predicted effects? Can we exclude the possibility that the global simulation of the whole brain simply provides a hyped-up robe of metadata, used to visualize more localized perturbation dynamics in a richer context? In my view, additional targeted *in situ* experiments are necessary to understand the real differences, and to learn from these before making sense of the alternative source of knowledge provided by the virtual experimentation paradigm. To what extent is it a valid funding strategy to encourage “virtualization” as a way to replace *in situ* experimentation?

Recently, the HBP consortium has answered to these criticisms by distancing its current objectives from the multiomic initial approach of the ramp-up phase of the project (Markram et al., 2011; Markram, 2012). In Section 6 of the white Zenodo paper generated at the end of the HBP flagship (Amunts, et al., 2023), the reformed consortium brings a clear semantic clarification: “We distinguish purpose-driven digital twins from the abstract idea of a full digital replica (or duplicate/copy) of the brain, the latter being the complete representation of all aspects of the brain at all levels. A full replica of the brain is neither achievable nor does it seem of clear practical use.” Note however, that HBP does not take the full responsibility for its repeated use of the “twin” metaphor in relation to a biological brain, whose journalistic media impact (the “twin” duplicate), will unfortunately prevail in the eye of the public.

### Building a unified multiscale theory of the brain

The global scientific strategy did change radically during the HBP flagship. At the start, “reverse engineering,” advocated by the charismatic Henry Markram, was the driving justification for the industrialization of neuroscience, the multiomic collection of big data and the need for high performance computing (HPC) infrastructures. One may agree or not with the soundness of the working hypothesis (see, for instance, Pitra, 2013; Frégnac, 2017), but it had the merit of providing a roadmap. Although doubtful about the Lego-like reconstruction strategy envisioned by Henry Markram, many of us thought that such an extensive reductionist exploration phase might uncover unsuspected alleys necessary for building a more comprehensive model of the brain.

The reductionist phase (Markram, 2012) progressively waned, when the triumvirate reduced experimental funding to the benefit of HPC, neuromorphic technology and future database infrastructures (Amunts et al., 2016, 2019), as if the community had siloed from the start enough low-level biological information to crack the brain. In the second part of the flagship, mean field (or neural mass) approaches and human brain imaging became dominant. In my view, this progressive shift in focus reflects the mid-flagship restructuration in governance, proposed by the Marquardt (2015) report, rather than a fundamental change in the way of looking at the brain. Science historians might make a finer diagnosis. Still, in a more scientific context, the shift could be interpreted as a down-grading of the initial ambitions, signaling the defeat of the theoretical field’s ambition to provide a multiscale view of the brain.

Let us expand a bit further. The main pragmatic issue of the reductionist phase during HBP was to decide how deep into the microscopic dimensions should the experimentation go. Unfortunately, we do not know yet. As early as 1714, the philosopher and mathematician Gottfried Wilhelm Leibniz argued that “if one could enter the brain as one enters a mill, there would be only mechanical parts, but one would not be able to observe thoughts” (cited in Cobb, 2020). Jonas and Kording (2017) confirmed the intuition of the “brain-mill” metaphor of Leibniz, by showing that reverse engineering methods would fall short of producing meaningful understanding of neural and computer systems, regardless of the amount of data. From the start of HBP, as well as in concurrent global neuroscience initiatives, a continuous debate has been whether reverse engineering is just a time/energy-consuming path, or a dead-end strategy (Pitra, 2013). Time will tell.

Following the transfer of the scientific leadership from EPFL to the Forschungszentrum Jülich, the HBP consortium gave less attention to molecular/cellular diversity, concentrating on brain imaging and mean field (neural mass) mesoscopic approaches. The mesoscopic dimension takes the lead in the public eye, mostly because striking correlations have been established between brain imaging mapping and perceptual or behavioral output. Even if fMRI signals are of a complex origin, mixing vascular, glial, and local field components rather than neuronal activity itself (Sirotin and Das, 2009; Schulz et al., 2012), they seem to provide topological markers of a medley of global activity. As such, they are used to encode the read-out of the holistic brain. This fits with what we know already from a phrenology-like localizationist approach (but with what statistical confidence? See Eklund et al., 2016; Murphy, 2016). This mapping strategy has been generalized with impressive success, to build, on the basis of more abstract labels, cognitive and semantic atlases (Huth et al., 2016).

In terms of computational neuroscience and theory, the HBP consortium has thus progressively shifted its focus of attention to the mesoscopic scale, to the detriment of pursuing an in-depth analysis of multiscale integration. The mean field formalism is attractive and seems the best candidate, since it provides a way to interpret brainwave diffusion in the context of functional brain maps. Its use has been most successful in understanding motor planning, decisional processes and speech production, all represented topographically in dedicated cortical output domains. Mean field models are also used to probe the dynamics of excitability states and their predictions guide cortical lesion experiments in epileptic patients.

However important hurdles persist. The mean field, or neural mass, certainly has global simplifying virtues (Deco et al., 2008; Pinotsis et al., 2014), but it relies on unproven assumptions of stochasticity and Gaussian distributions at more microscopic scales. The supposed stochasticity assumed by mesoscopic studies in fact ignores the biological diversity seen at the more microscopic level. This diversity is an essential functional specificity of central nervous system biology. Each molecular or cellular subelement is a carrier of retrievable information and its contribution to the mass (or, for the sake of illustration, columnar) activity field cannot be treated as purely additive noise or variability around a mean, as in traditional physics.

Other levels of complexification come with the multiscale organization of the living brain. For instance, efficient connectivity (functional connectome measures derived from fMRI resting state studies) in the brain does not depend only on the density of supposed connections and anatomic interaction distances (Mill et al., 2017). At a more microscopic level, functional wiring is specific to cell types and membrane affinities, it reflects the heterogeneity of local synaptic correlations and, very importantly, it is a dynamic function which depends on the timing-dependent integration of temporally structured factors and the history of past activity of the network. Consequently, smoothed (averaged) thresholded interpretation of fMRI studies leads to an interpretative view disconnected from the picture of integrative diversity obtained from results gathered in functional synaptic studies (Smith et al., 2013).

The same danger of disconnection may occur between mesoscopic and macroscopic scales, when the global geometry of the brain is taken into account. Recent work by Pang and colleagues, modeling large-scale brain activity (Pang et al., 2023), suggests that the macroscopic geometry of the brain may exert a more fundamental and stronger constraint on temporal “para”-synchronization dynamics than does the complex inter-regional mesoscopic connectivity (corresponding to the effective functional connectome). Knowledge of the connectome is not enough (Bargmann and Marder, 2013). It does not tell us much about how mental processes are generated, nor how different areas synchronize on wider scales. The real scientific challenge, which, in my view, would justify the HBP flagship dimension, is to account for the functional organization of the brain across all scales, beyond what can be charted topologically in a homeomorphic fashion on the 3D-cortical envelope of the brain.

We are far from establishing a comprehensive physical theory of the human brain. We still have to integrate conflicting observations at different scales. The success of mesoscopic approaches in the physics of inanimate matter is well established (“more is the same” in Kadanoff, 2009), but may not be strictly applicable to living glial and neuronal entities. Philip Anderson in his creative essay (“more is different”) described how new concepts, not present in ordinary classical or quantum mechanics, can arise from the consideration of aggregates of large numbers of particles (Anderson, 1972). With present knowledge, the system we study is necessarily a too complex product of evolution to be faithfully described by reduced equations. All we can do is to extract partial caricatures. These “simpler” representations can be used and manipulated to produce insightful predictions in specific contexts, but they may have, by construction and assumptions, eradicated important sources of causality and information. A new type of physics, of animated matter has to be invented, to better account for the multiscale stratification of information distributed in the living brain.

Consequently, and despite the claim of some HBP leaders (Amunts et al., 2019), integrating the three scales (micro-meso-macro) in a comprehensive functional model of the brain remains a challenge unsolved by HBP. Ten years later, we have still not decided whether spikes alone can yield useful simulations. We do not yet know if more microscopic variables and silent synaptic events, such as shunting inhibition, need to be considered, or whether dendro-dendritic computation may become effective in some globally ignited “conscious” states (Aru et al., 2023). Similarly, we suspect that more distributed or holistic information binding (glia-neuron communication, neuromodulation, nonsynaptic coupling effects; Cunha et al., 2022; Pinotsis and Miller, 2023) may be needed to account for the versatility and adaptivity of brain function. Perhaps now is the time to consider forming a more general quantitative model of brain networks not governed by statistical measures of association between spatial signals but based on the underlying most elemental physical properties of neural tissues from which those signals emerge (Van Horn et al., 2023).

The complexity of the model needed to accommodate the ever-growing flow of produced data may become commensurate with that of the brain itself (Borges, 1946)! In brief, simulating the full brain remains a target that is out of reach. Running simulations of its simplified caricature is certainly of major interest, but it should not make us forget that such models are not “twins” of the brain. As Steve Jobs, the founder of Apple, used to say: “Details matter. It’s worth waiting to get it right.”

## Transforming Criticism into a Proactive Agenda

An ideal overview of the flagship experience should not create a divide between those who do the hard work and those who criticize, and stop us from modeling the brain! It should provide the opportunity to open a constructive dialogue and suggest alternative pathways. Crossing the finish line of HBP, we are now in a better position to identify the bottlenecks, characterize which objectives were not met, and find the causal reasons which limited the success of the flagship. By turning these around, we can transform them into proactive proposals (what SHOULD or COULD have been done). By applying lessons from the three areas of scientific planning, community coordination, and management, can we define what alternative strategies might have better served the steering of such an ambitious flagship?

Box 1. Scientific planning

- Rather than simulating the whole brain, science in the flagship should have been focused on a selected number of “hard problems,” validated by the scientific community, and for which the need for a collective interdisciplinary synergy could be demonstrated.
- The “flagship” dimension should enter in through the blue-sky prospect of a unified theory of the brain, linking structure and function, across spatial and temporal scales, in a causative and predictive way.
- Ideally, to make collaboration effective, the number of teams – each selected for an innovative combination of technology or expertise for a given task, should be medium size (10–20), with each team composed of 5–20 labs or experienced ERC-funded researchers. A case-example of such an effective team exists already: The International Brain Laboratory (2017) regroups with impressive success twenty-two self-selected labs and fifty PIs around a single basic task in the behaving mouse, in a comprehensive project designed to probe decisions based on visual perception and on history of reward. The present view further includes the coordination of teams and tasks at a macroscale level, adding further coherence to the collective agenda and partnership, independent of individual merit.
- Most importantly, the flagship should include an “integrator” consortium of interdisciplinary labs and theoretical centers, with a full-time focus on the final integration of the flagship research findings. The aim would be to produce a strong theoretical framework that generates testable predictions for the future.

To feed the model(s), the whole community should first agree on common standards of data and metadata. Significant funding should be devoted to ensuring that new datasets and relevant existing datasets are interoperable. Consolidated databases should be open and not privately owned. When possible, theoreticians should partner with the experimenters from start in defining the dataset which will feed their simulations. All new sets of data should be acquired using agreed-on shared criteria. Planning should include comprehensive think-tank initiatives, fostering participation from the widest audience.

Comparative animal experimentation studies should be encouraged, to better understand commonalities and distinctions between humans and other species. Species should be chosen according to the studied behavior and cognitive capability, and not solely based on the technology availability. If the necessary technologies do not exist for a species, the development of the appropriate tools should be part of the roadmap and should precede the functional and behavioral experimentation.

Across-scales biophysical integration studies should be systematically developed, to help with data integration and theoretical hypothesis testing. Research objectives should not be confined to fitting performance, but should be structured to identify causative internal neural mechanisms.

Major efforts should be made in modeling strategies and brain theory. Scientific exchanges should be reinforced by all means, and should benefit from the existing structures in the theoretical field (Europe: Bernstein Centers, Gatsby, EITN; United States: Kavli Foundation, Simons Institute; International: INCF, as examples). Permanent facilities should be created in key scientific nodes, taking the form of Institutes of advanced studies, to increase the effectiveness of interdisciplinarity and state-of-the-art training of young scientists. European courses in theoretical/computational neuroscience should be organized on a yearly basis (EITN, Bernstein), in close coordination with related international initiatives (e.g., United States: Tellurides, Cosyne, Cold Spring Harbor Laboratory (Woods Hole); Japan: Riken-BSI; China: Cold Spring Harbor Asia, ION) and the EC initiative in neuromorphic engineering (CCN).

Box 2. Community coordination

- The targeted research and industry communities should be clearly defined from the start.
- Funding should be guaranteed for 10 years, and renewable in the case of tangible success. This extended duration provides tranquility and the capacity to think of long-term impact. Note that renewed funding has been achieved for the Graphene flagship, while the support necessary for the viability of EBRAINS remains somewhat modest and still under scrutiny.
- The EC funding priorities should take the form of targeted support of mid-size consortia (to ensure effective collaborations) and ERC-funded individuals (to sustain original proposals). These should be registered and coordinated in the global roadmap context.
- Priorities should also include the production of appropriate tools for the community to ensure interoperability between the databases and models produced by the various actors. Note that this issue motivated the creation of EBRAINS as a tool and infrastructure provider following HBP.
- This coordination would require, from the start, the active and direct participation of the main national and federal research agencies (e.g., CNRS, Max Planck Gesellschaft, DFG, CNR) and private centers already funding scientific excellence (e.g.,, International Brain Laboratory, ELSC among many), as well as the main European scientific coordinating networks (e.g., Bernstein Network). Note here that, in contrast to the HBP, this coordination strategy was fully operational from the start of the United States BRAIN initiative.
- Collaborations and Interdisciplinarity should be encouraged through extrafunding initiatives (see for instance the “Collaborative Research Center” funding program (CRC) by the German Research Foundation to reinforce collaborations and the innovative “Change of Course” program of the DFG/Volkswagen Foundation).
- The growth in interdisciplinary and effective networking of the community should be monitored, using social sciences metrics.
- The same scientific experts should follow a flagship’s progress throughout the entire project duration.
- Education (including Ethics) should play a much stronger (and better funded) role in all activities, since flagships go further than teaching tools, by also building a culture and a way of thinking/operating (see for instance the success of Marie-Curie FACETS-ITN in earlier FET initiatives). This will take time over several successive flagship durations.

Box 3. Administration and management

- The scientific flagship leadership should be governed by an interdisciplinary and decision-making board of scientific directors (not by administrators). These directors should be representative of their fields of expertise, and should accept to spend a sizeable fraction of their professional activity on flagship issues, and be free of conflicts of interest.
- Management should capitalize on existing administrative resources at the participating institutions. Administration should be delocalized across the main European centers and shared by existing national institutional agencies and private centers of excellence, requiring already proven their efficiency in coordinating science and willingness to work in concert. To ensure their participation, a stable rate of recurrent funding should be guaranteed, with the appropriate safeguard monitoring measures.
- Hiring policy should make use of the exceptional long time course of the flagship (10 years). To ensure scientific continuity throughout the project, long-term recruitments to key roles should be made possible in all participating countries.

## Conclusion: Virtual Replay

Thanks to the Human Brain Project, we have been given an exceptional opportunity to advance knowledge in brain science and to change science and technology around the brain. The results are what they are.

Some may be tempted to stop the tide of funding to better see whether a strong legacy will survive. The future of EBRAINS will be closely linked to the use that the scientific community will make of the infrastructure that HBP finally delivered. A pause period could also be used to realign HBP efforts with the other global neuroscience initiatives, and perhaps to transfer the responsibility or leadership to other national or international institutions, mature enough to persist in the quest. However, a pause, if it happens, should be transient. In my view, flagship projects like HBP are needed because they raise society awareness on fundamental questions: how should science evolve (data-driven or hypothesis driven, big or small, or both); how should technological races and promises of industrial profit influence (good or bad) the way we think about fundamental issues and complex scientific objects, such as the brain; what part should societal applications should take in defining (fostering or reasoning) flagships to fulfill our blue-sky ambitions?

The possibility of a second flagship shot, in the field of brain sciences and neurotechnology, is at present unlikely in the present format, at least in Europe. Opening a new window in the near future would require at least three changes in the conception of flagships, involving all the community leaders, scientists, eurocrats and industrialists. We need: (1) greater modesty, in view of the complexity of the brain; (2) honesty and acuity, in identifying the bottlenecks met by the previous flagships; and (3) proactive inspiration from our successes and failures. In my view, the justification for renewing flagships must depend on the acuity and reactivity with which we, as a community, are able to learn lessons from past experience and adapt our way of doing science.

Fifty years ago, after the exhilarating hopes generated by the rise of strong artificial intelligence (AI) and symbolic computability using Turing machines, the “Lighthill report” (1973), commissioned by the British Science Research Council gave a very pessimistic prognosis, stating that “in no part of the field have the discoveries made so far produced the major impact that was then promised.” As neuroscientists passionate about the “Mind,” it remains our collective responsibility to avoid a “winter” period, already experienced when the “Computer” suddenly became the “tenor” (Richards, 1936) of the computational metaphor of the brain. Such winters occur when the promises are too high.

Let the “Brain” remain the “tenor,” and IT the “vehicle.” Counter intuitively, it could be that the sense of incompleteness, if not frustration, experienced at the end of the first act of HBP puts scientists in the right mind, to consolidate a coherent roadmap and clear targets with guidance based on theory from the start. We need to recognize the limits of caricatures seeking to account for biological complexity, to conceive more rigorously the engineering of task-specific digital twins (in the industrial neuro-IT sense) as applications, and to avoid pretending that macroscopic phenomenology explains how the real brain works. Why? Because this first edition of the flagship has given scientists significant time to find out (1) what does not work and (2) what matters. More importantly, more scientists may now fully recognize the importance of building an effective community and a collective agenda.

So, let us be more optimistic and dream of a possible replay of HBP: “Please, play it again, Sam…” Of course, this virtual closing note and meme is directed neither at Sam the pianist playing “As Time Goes By” in the movie *Casablanca*, nor at Henry and Katrin from HBP. It simply expresses my own desire, as a nostalgic scientist, to go back in time, to re-establish a lost relationship, and restart a new history, with greater hope…

## Synthesis

Reviewing Editor: Christophe Bernard, INSERM & Institut de Neurosciences des Systèmes Decisions are customarily a result of the Reviewing Editor and the peer reviewers coming together and discussing their recommendations until a consensus is reached. When revisions are invited, a fact-based synthesis statement explaining their decision and outlining what is needed to prepare a revision will be listed below. The following reviewer(s) agreed to reveal their identity: NONE.

na
